# The Impact of Organic Matter on Polycyclic Aromatic Hydrocarbon (PAH) Availability and Persistence in Soils

**DOI:** 10.3390/molecules25112470

**Published:** 2020-05-26

**Authors:** Aleksandra Ukalska-Jaruga, Bożena Smreczak

**Affiliations:** Department of Soil Science Erosion and Land Protection, Institute of Soil Science and Plant Cultivation—State Research Institute, Czartoryskich 8, 24-100 Puławy, Poland; bozenas@iung.pulawy.pl

**Keywords:** PAHs, bioavailability, persistent contaminants, soil organic matter, fulvic acids, humic acids, humins

## Abstract

Polycyclic aromatic hydrocarbons (PAHs) exhibit persistence in soils, and most of them are potentially mutagenic/carcinogenic and teratogenic for human beings but also influence the growth and development of soil organisms. The PAHs emitted into the atmosphere are ultimately deposited (by dry or wet deposition processes) onto the soil surface where they tend to accumulate. Soil organic matter (SOM) plays an important role in the fate and transformation processes of PAHs, affecting their mobility, availability, and persistence. Therefore, the aim of this research was to investigate the influence of SOM fractional diversification (fulvic acids—FA, humic acids—HA, and humins—HN) on PAH availability and persistence in soils. Twenty soil samples (*n* = 20) were collected from upper horizons (0–30 cm) of agricultural soils exposed to anthropogenic emissions from industrial and domestic sources. The assessment of PAH concentrations included the determination of medium-molecular-weight compounds from the US EPA list: fluoranthene—FLA, pyrene—PYR, benz(a)anthracene—BaA, and chrysene—CHR. The assessment was conducted using the GC-MS/MS technique. Three operationally defined fractions were investigated: total extractable PAHs (TE-PAHs) fraction, available/bioavailable PAHs (PB-PAHs) fraction, and nonavailable/residual PAHs (RE-PAHs) fraction, which was calculated as the difference between total and available PAHs. TE-PAHs were analyzed by dichloromethane extraction, while PB-PAHs were analyzed with a hydrophobic β-cyclodextrin solution. SOM was characterized by total organic carbon content (Turin method) and organic carbon of humic substances including FA, HA, HN (IHSS method). Concentrations of PAHs differed between soils from 193.5 to 3169.5 µg kg^−1^, 4.3 to 226.4 µg kg^−1^, and 148.6 to 3164.7 µg kg^−1^ for ∑4 TE-PAHs, ∑4 PB-PAHs, and ∑4 RE-PAHs, respectively. The ∑4 PB-PAHs fraction did not exceed 30% of ∑4 TE-PAHs. FLA was the most strongly bound in soil (highest content of RE-FLA), whereas PYR was the most available (highest content of PB-PYR). The soils were characterized by diversified total organic carbon (TOC) concentration (8.0–130.0 g kg^−1^) and individual SOM fractions (FA = 0.4–7.5 g kg^−1^, HA = 0.6–13.0 g kg^−1^, HN = 0.9–122.9 g kg^−1^). FA and HA as the labile fraction of SOM with short turnover time strongly positively influenced the potential ∑4 PAH availability (r = 0.56 and r = 0.52 for FA and HA, respectively). HN, which constitutes a stable fraction of organic matter with high hydrophobicity and poor degradability, was strongly correlated with ∑4 RE-PAHs (r = 0.75), affecting their persistence in soil.

## 1. Introduction

Polycyclic aromatic hydrocarbons (PAHs) are contaminants of global concern, and in soils they can be found at high concentrations, indicating a potential environmental hazard. PAHs mainly originate from the long-term deposition of airborne particles emitted from natural (e.g., volcanic eruptions, forest fires) and anthropogenic (e.g., industry, traffic, road runoff) sources [1,2,3,4,5,6,7,8]. These contaminants are susceptible to global long-range transport; therefore, they can be detected in soils even at long distances from their emission sources [9].

PAHs belong to the group of hydrophobic organic contaminants (HOCs), indicating persistence in soils and affinity for accumulation in living organisms. Some compounds from this group (e.g., benzo/a/pyrene) are well known as mutagens, carcinogens, teratogens, and endocrine disruptors (International Agency for Research on Cancer, 2010). Occurring at high concentrations, they can also influence the activity of microorganisms and/or the growth and development of plants and invertebrates [7,10].

PAHs present in soils undergo various processes that lead to their chemical transformations (photolysis, oxidation, hydrolysis), biological degradation and uptake as well as volatilization and leaching, sorption to various soil components, diffusion, and partitioning between different soil compartments. These processes are influenced by biological, chemical, and physical properties of soil, climatic factors, the soil–contaminant contact time, and the properties of contaminants [3,6,7,9,10,11,12,13]. The net output of these transformations over a long period of time causes changes in PAH content and their availability to soil biota.

In recent decades, scientific efforts were dedicated to the testing and application of chemical methods to determine the PAH available fraction as an important measure in the risk assessment of contaminated areas. A potentially available PAH fraction is operationally defined as containing compounds dissolved in soil solution and readily desorbing into a liquid phase. Numerous experiments confirmed the statistically significant positive relationship between the quantity of potentially available PAHs and reactions of living organisms, predominantly microbial activity [7,14,15,16].

Soil organic matter (SOM) is a significant factor affecting the behavior of organic contaminants in soils [9,11,12,17,18,19,20,21,22,23,24]. According to Pignatello [19], the mechanism of PAH sorption to organic matter can be caused by van der Waals attraction, hydrophobic bonding, hydrogen interactions, charge transfer, intermolecular forces, or ligand exchange. PAH sorption by SOM is the major process that controls the partitioning of these compounds causing the strong retention (aging) of hydrocarbons and limiting their diffusion or release into the soil solution [9,18,20,22].

SOM is a collective term for all soil organic materials and contains various components, mainly humic substances, kerogen, bitumen, and black carbon [9,18,20,22,24,25,26,27]. Different soil organic matter components vary remarkably in structure and composition, and therefore exhibit deviated sorption properties to organic contaminants [24,25,26,27,28]. SOM is a nonhomogeneous and complex mixture of macromolecular compounds of plant and animal origin at various decomposition stages, as well as humic substances (HS) of specific properties [25,26,27]. According to the classic approach [26,27], HS have been classified into three main fractions, fulvic acids—FA, humic acids—HA and humins—HN, characterized by diverse physicochemical properties, structures, and sorption affinities [19,29,30]. The bases of the structure of humic substances are aliphatic chains or aromatic rings containing various functional groups (e.g., -COOH, -OH, =C=O) that are responsible for chemical reactivity [25,26,27,28]. Molecular sizes, chemical structures, redox-active functional groups, complexing sites, aromaticity, hydrophobicity, and polyelectrolytic characteristics depend on the individual fractions of humic substances and affect their affinity for binding to the contaminants [28]. FA and HA are relatively soluble in soil solution, while HN are insoluble in water and create a mixture of hydrophobic compounds like lignins, waxes, fats, and other aromatic components. 

Numerous studies have been carried out on areas polluted with PAHs, but the results referring to the SOM–PAHs relationship varied substantially. The statistically significant positive SOM influence on PAH content was also reported. These findings indicate that persistence and availability of hydrophobic compounds may be governed by SOM fractional composition rather than its total content. Therefore, the aim of this study was to investigate the influence of SOM fractional diversification (FA, HA, HN) on PAH potential availability and persistence in soils subjected to natural environmental variables (e.g., precipitation, temperature, intra-soil processing).

## 2. Results and Discussion

### 2.1. Assessment of the Concentrations of Total Extractable PAHs (TE-PAHs), Potentially Available PAHs (PB-PAHs), and Residual PAHs (RE-PAHs) and Their Mutual Relationship

The concentrations of ∑4 TE-PAHs were strongly diversified throughout the sampling area (Table 1) and ranged from 193.5 to 3169.5 µg kg^−1^. In more than half of the samples the ∑4 TE-PAH content was above 1000 µg kg^−1^, which was also the median value. The average value (1120.2 µg kg^−1^) was close to the median value (1065.1 µg kg^−1^), indicating a normal distribution of the results. All compounds showed a level of variability within the range from 56% to 99%. Nevertheless, the detected concentration of ∑4 TE-PAHs was similar to data from former industrial sites of France [31,32], Czech Republic [33], Norway [34], and significantly lower than in China [35] or Japan [22]. For individual four-ringed compounds, the average highest content was found for fluoranthene (FLA; 486.7 µg kg^−1^, within the range from 84.6 to 1220.1 µg kg^−1^) and pyrene (PYR; 367.9 µg kg^−1^, within the range from 50.3 to 948.3 µg kg^−1^), while the lowest was detected for benz(a)anthracene (BaA; 152.3 µg kg^−1^, within the range from 15.4 to 725.8 µg kg^−1^), as shown in Table 1. The permissible content of BaA (100 µg kg^−1^) and chrysene (CHR; 200 µg kg^−1^) in agricultural soils according to the Polish national regulations (JoL 2016 item 1395 [36]) was exceeded in 70% and 30% of analyzed samples, respectively.

**Figure 1:**
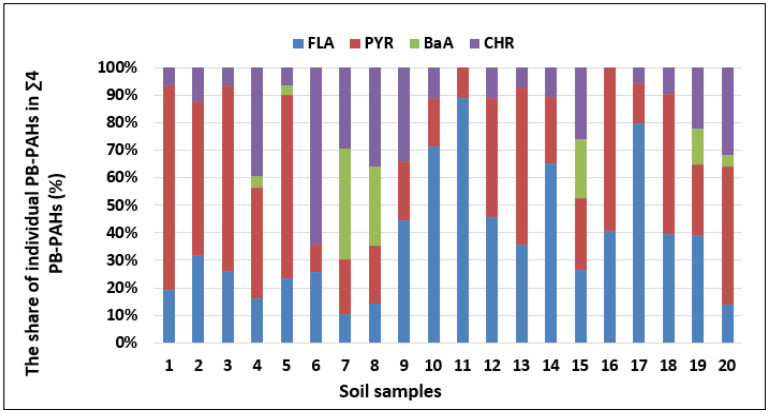
The share of the potentially available fraction of individual compounds in the TE-∑4PAHs determined in soil samples (*n* = 20).

The highest concentrations of the available PAHs fraction were recorded for PYR (18.1 µg kg^−1^) and CHR (16.2 µg kg^−1^), while lower concentrations were recorded for FLA (14.2 µg kg^−1^) and BaA (7.8 µg kg^−1^), as shown in Table 1. The PB-PAHs (PB-FLA, PB-PYR, PB-BaA, PB-CHR) accounted for 0.2%–29.4% of TE-PAHs (TE-FLA, TE-PYR, TE-BaA, TE-CHR) with a high diversity of results (coefficient of variation, CoV, was at the level of 103% to 183% for individual compounds, Table 1, Figure 1). The potentially available fraction of FLA, PYR, BaA, and CHR did not exceed 1% in almost 25% of samples, while 15% had a total content of individual PAHs that was higher than 20%. The obtained results indicated that despite the relatively high total content of individual PAHs in soils, the content of their available fraction is relatively small, which indicates a low risk of their transfer to living organisms and groundwater.

The contents of the residual fraction of PAHs (RE-PAHs) was very closely related to the total concentrations of these compounds (Table 1) due to the low PB-PAH content. Generally, ∑4 RE-PAHs constitute between 71% and 99% of ∑4 TE-PAHs, with the strongest retention of RE-FLA (84.3%–99.2% of TE-FL) and RE-BaA (58.2%–99.9% of TE-BaA) in soils. Similar results were obtained by Sun et al. (2013), Barnier et al. (2014), Sánchez-Trujillo et al. (2014), Cachada et al. (2014), Kołtowski et al. (2016), and Smreczak (2018), who analyzed the total and potentially available concentrations of PAHs in soils from industrial areas subjected to long-term anthropopressure. A large array of chemical and physicochemical methods applied for the measurement of the availability of PAHs does not enable proximate comparison of the results because the final concentration of the PB-PAHs fraction results from the extraction procedure [32,37]. The methods involved either a mild organic solvent extraction or application of cyclodextrins or solid-phase hydrophobic adsorbents such as XAD resin or Tenax-TA [7,10,12,13,32,38,39,40,41,42]. Regardless of the applied method, the quantity of available PAHs was significantly lower compared to the total concentration that indicated the low aqueous solubility of PAHs and/or their strong sorption on soil components and partitioning among various phases [14,15,16]. Cyclodextrin extraction was firstly identified by Reid et al. [38] and then confirmed by other authors [13,31,39,41,43] as a suitable technique that recognizes the readily desorbing fraction available for biota, causing a real risk in the contaminated areas.

The TE-PAH compounds were not significantly correlated with PB-PAHs, which may indicate the historical deposition of hydrocarbons and occurring aging processes. Some authors observed significant positive relationships indicating that soils with a higher concentration of TE-PAHs were characterized by a higher content of PB-PAHs [13,39]. Nevertheless, the research was conducted in soils artificially fortified by these compounds in laboratory conditions. Availability of PAHs in these soils was quite high due to a short incubation time (from one day to several months) that influenced aging processes [40,42]. In the natural soil environment, many different factors may affect PAH behavior, which is mainly associated with the repetitive depositions of contaminants and strong sorption of these pollutants by various soil components occurring over a long period of time as well as diffusion of PAH molecules within structures of organic matter [3,7,11,12,14,15,16,32,40,41,42]. The importance of the aging processes was confirmed by the high share of the RE-PAH fraction of individual compounds.

### 2.2. Assessment of Soil Organic Matter (SOM) and Its Fractional Composition (FA, HA, HN)

**Figure 2:**
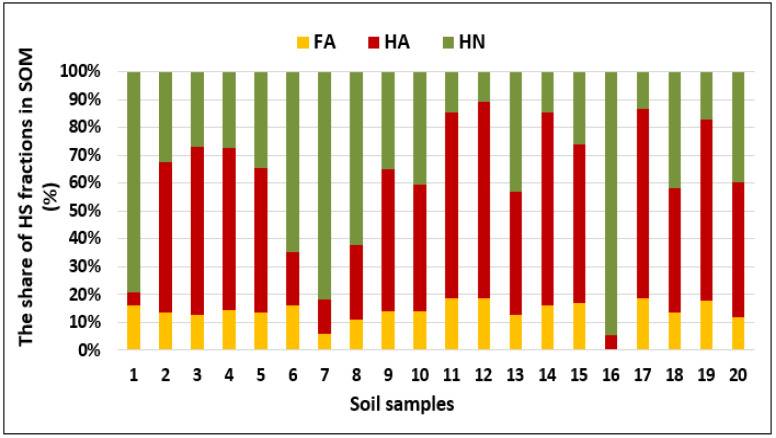
Composition of HS fractions and their percentage share in the total soil organic matter (SOM) content (*n* = 20).

SOM content in the analyzed soils exhibited typical values for Polish soils of agricultural usage [44] and was in the range 8.0–130 g kg^−1^ (CoV = 120%) with a median value of 13.8 g kg^−1^. SOM was fractionated onto individual HS components, the content of which was not dependent on the total content of organic matter (Table 2). HS was highly diversified, and among the compounds, HN constituted the most heterogeneous fraction (CoV = 192%) compared to FA (CoV = 68%) and HA (CoV = 43%). Similarly, HN constituted a significant portion (0.9–122.9 g kg^−1^) of humic substances, while more labile fractions of humic acids occurred in the range from 0.4 to 7.5 g kg^−1^ and 0.6 to 13 g kg^−1^ for FA and HA, respectively. A general share of individual fractions (in total SOM content) followed the overall trend of HN (10.3%–94.5% of total organic carbon—TOC) > HA (4.6%–66.4% of TOC) > FA (0.3%–17.7% of TOC), as shown in Figure 2.

The mutual proportions between HA and FA were above one for all soil samples (HA/FA = 1.0–18.2 with a median value of 3.7), while the ratio of (FA + HA)/HN was between 0.1 and 8.1 (with a median value of 1.9), as shown in Table 2. The proportions between HA and FA fractions are expressed as HS transformations and indicate the potential low mobility of C in the soil system and a high intensity of humification processes, while the proportion of (HA + FA)/HN shows the degree of organic matter transformation to the stable organic matter forms [24,45]. Higher values of the above two parameters suggest a lower mobility of organic matter and its weaker susceptibility to mineralization. 

### 2.3. Influence of SOM on Concentrations of Total Extractable PAH (TE-PAHs), Potentially Available PAHs (PB-PAHs), and Residual PAHs (RE-PAHs) in Soil

The main factor controlling the PAH content in soils is the SOM and its fractions [5,9,10,11,12,17,18,19,20,21,22,23,24], as shown in Table 3. Although the TOC content does not affect directly the concentration of the potentially available PAHs fraction, a higher organic carbon content generally results in increased sorption of PAHs and a reduction of their accessibility. It is assumed that soils amended with exogenous organic matter increase pollutant sorption and reduce their absorption by living organisms. Many different studies have been conducted in this field [11,12,21,23]. 

**Figure 3:**
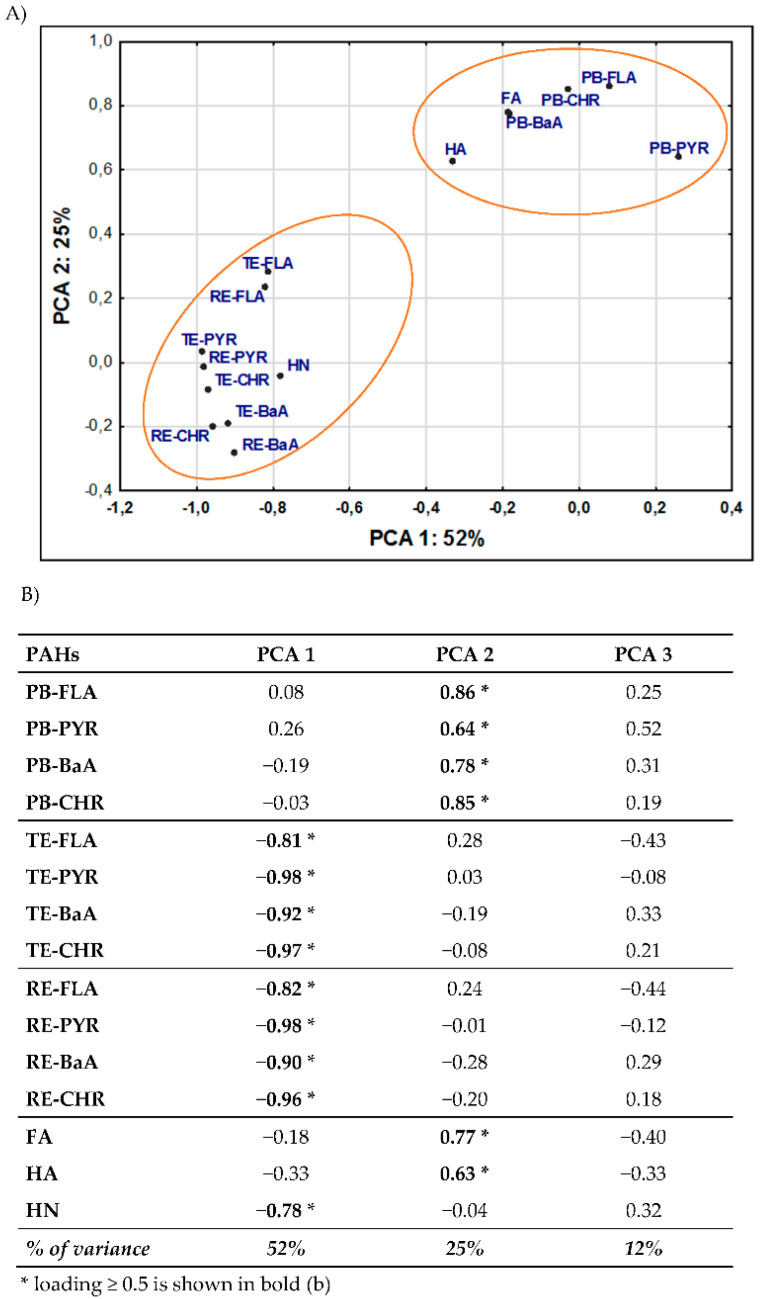
PCA: ordination biplot (Component 1 vs. Component 2) used to generate the PC components describing the impact of the HS fractions on the content of PAHs for all analyzed data sets (*n* = 20). Points in the plot represent variable loadings relative to each component (**A**). The table of significant factor loadings and the matrix were determined based on the scree plot (**B**).

The origin and SOM composition can profoundly influence the association and immobilization of PAHs. Different fractions of organic matter bind PAHs with different strengths and affect their half-life and resistance in soils [18,19,20,21,22,24]. Results of correlations between PB-PAHs, TE-PAHs, and RE-PAHs and FA, HA, and HN clearly indicate that availability of contaminants is promoted by labile humic substances (FA, HA), while persistence depends on the stabile carbon forms (Table 3, Figure 3). Nevertheless, the impact of individual SOM fractions on the PAH behavior may be also controlled by the properties of the contaminants and their affinity with the organic soil phase, expressed by the K_o/w_ coefficient (Table 5).

According to correlation results, FA and HA exhibited significant positive correlations with ∑4 PB-PAHs (r = 0.56 and r = 0.52, respectively), while ∑4 TE-PAHs were strongly positively correlated with HN (r = 0.70), as shown in Table 3. HN also significantly influenced ∑4 RE-PAHs (r = 0.75); however, this resulted from the prevalent share of ∑4 RE-PAHs in the total content of these compounds. Among all PAHs, FLA indicated highest sorption affinity with FA and HA, regardless of the PAH operational fraction (r = 0.50 and r = 0.56 for PB-PAHs; r = 0.57 and r = 0.59 for TE-PAHs; r = 0.50 and r = 0.56 for RE-PAHs). The PYR and BaA occurring in potentially available forms were strongly connected with HA (r = 0.55 for PB-PYR and PB-BaA) in contrast to CHR, which was more strongly associated with FA (r = 0.73 for PB-CHR). The obtained dependencies indicate that the higher contribution of humic acids (FA, HA) to total SOM content caused the greater availability and potential mobility of hydrocarbons in the soils, while high amounts of HN affected their higher stability.

Moreover, the processes of SOM transformations expressed by HA/FA proportions significantly positively influenced the total and residual concentration of PAHs (r = 0.50–0.82 for individual TE-PAHs and r = 0.51–0.84 for individual RE-PAHs). This means that the transformation process of organic matter constitutes an important factor in the accumulation of PAHs in agricultural soils from areas under anthropogenic pressure. The more advanced the humification processes, the greater persistence and lower potential availability of PAHs.

For a more detailed recognition of the relationship between the effect of the SOM fractions on the PAH content, PCA analysis was used. All analyzed data are represented by three main components, explaining 89% of the total variance of the results (Figure 3A), whereas up to 77% of variance is explained by the first two factors (Figure 3B). The first PCA component (PCA 1), which accounted for 52% of variance, was significantly negatively correlated with individual TE-PAHs and RE-PAHs (r = −0.81 to r = −0.98 and r = −0.82 to r = −0.98, respectively), while the second PCA component (PCA 2), which represented 25% of the results’ variability, was positively correlated with PB-PAHs (r = 0.64 to r = 0.85). All variables are well represented by the first two main components of the PCA coordinates, but the impact of the third PCA component (PCA 3) reached almost 12%. Although the loads were not correlated with any of the analyzed variables, its share may be related to the chemical properties of hydrocarbons or other undefined processes or properties affecting the behavior of PAHs in soils. These results more clearly indicate that individual SOM fractions determine the accumulation of PAHs and their availability in soils. PAHs were divided into two independent groups, and the distance between variables confirmed a high mutual relationship pointing to significant soil parameters affecting their contents. 

The results clearly showed (Table 3 and Figure 3) that in soils subjected to long-term anthropopressure, aging processes of the pollutants prevail. PAHs directly deposited in soils are subject to an “aging process”, which often entails an initially rapid and reversible sorption process to SOM followed by a period of slow diffusion occurring over weeks, months, or even years once they are released into the soil environment [15,16,17]. This indicates stronger PAH sorption by stable SOM structures and the persistence of PAHs in soil. During aging, contaminants persevere in soils and become increasingly recalcitrant to desorption and thus much less available, possessing lower bioaccumulation and biodegradation affinity or toxicity [16,17,19,20,31,32,38,39]. Therefore, the resistant fraction content increases with time, indicating significant shares of RE-PAHs in the total PAH content.

Aging involves a number of mechanisms but is predominated by diffusion into micro- or nanopores [16,17] and partitioning into soil organic matter [18,19,20,22]. It is generally thought that PAH sorption to SOM is influenced by two major domains, “soft/rubbery” and “hard/glassy” domains. Wang et al. [9] proposed the theory of sequential bonding of pollutants by SOM, which involves the following steps: Step 1, capturing the fraction of fresh pollutants through rubber FA (weak hydrogen bonds with PAH, facilitating the migration in the soil profile); Step 2, binding through HA (metastable sorbent states, inclusion of HA micellar forms); and Step 3, slow sorption of contaminants into the glassy HN structures (process aging). The noted significant positive correlations between HN and RE-PAHs as well as FA, HA, and PB-PAHs (Table 3) confirm the migration path of hydrocarbons between SOM fractions.

Luo et al. [20] have suggested that surface characteristics of SOM fractions play a critical role in interactions with organic contaminants due to better access to the surface sorption/retention domains in the first stage after they are released into the soil. This implies that the abundance of surface carbon domains of SOM fractions plays an important role in attracting PAHs and in their further interactions. According to Schaumann [29,30] and Pignatello [19], the “soft/rubbery” and “hard/glassy” phases interpenetrate in the HS structure. HA and FA are mainly of amorphic nature with mostly “soft/rubbery” phases, which very quickly interact with contaminants, but the strength of the bond is weak. HN are characterized by higher condensation of structure (benzene rings and aliphatic chains) and thus are more abundant in “hard/glassy” phases, and sorption on their structures occurs slowly, but the sorbed molecules are relatively persistent [19,29,30,46]. The “hard/glassy” phase forms regions of disordered side chains, which have a high density and low reactivity and flexibility due to the presence of numerous unsaturated bonds [29,46]; therefore, they have a high capacity for retaining PAHs by diffusing them into the tertiary structure. The structure of the condensed and rubbery phase results in more active and stable binding sites for PAHs, which allows the effective retention of these compounds in soils [17,29,30]. Although general information about chemical and physical differences between various organic matter fractions have been previously analyzed [17,18,19,22,24,29,30], the operational separated humic substances may have other specific properties that promote their interactions with PAHs.

Generally, the mechanisms of PAH partitioning during aging are thought to involve the movement of PAHs to more recalcitrant binding sites, e.g., micropores that are too small for microbial access or deeper penetration into organic matter by encapsulation (occlusion) or strong sorption by SOM components [19,46]. The positive correlation between RE-PAHs and organic matter may confirm this. Diffusion of contaminants within organic matter structure and soil micropores may also result in their migration to the more “relaxed regions”, where the desorption of molecules to the soil solution is easy, or to regions where they are weakly bound by organic matter that induces the release of contaminants, resulting in an increase in the available PAH content.

## 3. Materials and Methods

### 3.1. Site Description and Sample Collection

**Figure 4:**
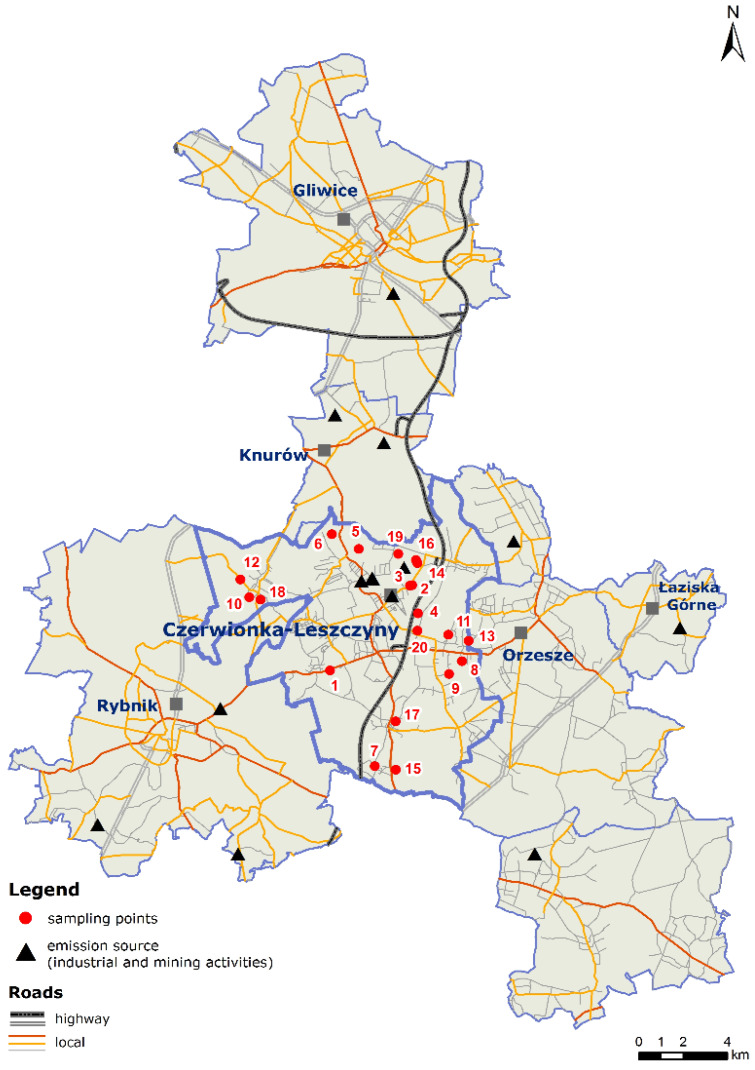
Location of the research area and the sampling points (*n* = 20).

The SOM and its fractions (FA, HA, HN) as well as the individual 4-ringed PAHs were isolated from soils subjected to long-term and strong industrial anthropogenic pressure (since 1910 when mining activity began on the study area). The sampling points (*n* = 20) were located in the Czerwionka-Leszczyny municipality (area: 115 km^2^; population density: 366 persons km^−2^; total dust emission: 6827 kg year^−1^ km^−2^; dust emission from industrial sources: 1084 kg year^−1^ km^−2^) situated in southwestern Poland (Silesia Voivodeship, Figure 4). The research area is covered by soils of agricultural, urban, and industrial usage belonging to the Rybnik Industrial District.

The main contamination sources of soils from the Czerwionka-Leszczyny area derived from fuel combustion, road transport, and industrial activities predominantly related to the disposal of environmentally harmful mining waste and production of bituminous masses. Industrial activity affected the strong transformation of the research area, resulting in the occurrence of numerous waste dumps and storage sites for metallurgical wastes. Moreover, the single-family housing clusters were an additional source of local domestic PAH emissions. A broader description of the study area is available in the publication of Klimkowicz-Pawlas et al. [5] and Ukalska-Jaruga et al. [24]. 

The soil samples were collected from the surface layer (0–30 cm) of agricultural soils situated at different distances and locations from potential PAH emission sources that influenced the concentration of these compounds and their contaminant–soil contact time (aging). The location of the sampling points (Figure 4) reflected the variability of soil physicochemical properties, which may influence differences in SOM content and its fractional composition (Table 4). The samples were properly prepared prior to chemical analysis by air-drying, passing them through a 2-mm mesh sieve, and storing them in the dark at 12–16 °C.

### 3.2. Determination of Soil Basic Physicochemical Properties 

The selected measured soil characteristics included: pH in 1 mol KCl, clay (φ < 0.002 mm), silt (φ = 0.002–0.05 mm), sand (φ = 0.05–2.0 mm), total nitrogen (TN), total carbon (TC) and total organic carbon (TOC) contents. The pH was measured potentiometrically in a 1:2.5 (m V^−1^) soil suspension in 1 mol L^−1^ KCl solution (PNISO10390, 1997). The clay content was analyzed via the aerometric method (PN-R-04032, 1998), while TN and TC were determined in a Vario Macro Cube CN elemental analyzer (Elementar Analysensysteme GmbH, Langenselbold, Germany) after dry combustion. TOC content was determined after sulfochromic oxidation followed by titration of the excess K_2_Cr_2_O_7_ with FeSO_4_(NH_4_)_2_SO_4_·6H_2_O (PN-ISO 14235, 2003).

### 3.3. Determination of PAHs 

Three operationally defined PAH fractions were investigated in the study: (i) the total extractable PAHs fraction (TE-PAHs), including compounds extracted by the intensive extraction technique using organic solvents; (ii) the available PAHs fraction (PB-PAHs), which refers to the fraction of contaminants that is biologically available (bioavailable) for soil organisms, present in soil solution, and may be readily desorbed from soil under the influence of a complexing agent; and (iii) the residual/(nonavailable) PAHs fraction (RE-PAHs), which describes the bound fraction of contaminants and was calculated as the difference between the total and potentially available PAHs (RE-PAHs = TE-PAHs − PB-PAHs).

Determined PAHs included 4-ringed compounds fluoranthene—FLA, pyrene—PYR, benz(a)anthracene—BaA, and chrysene—CHR, classified as medium-molecular-weight hydrocarbons (m/z 202 and 228) and indicated as pollutants of environmental concern on the US EPA list (Table 5). The selected PAHs exhibit various chemical properties, i.e., spatial conformation, molar mass, water solubility, vapor pressure, and the values of the octanol/water partition coefficient. Moreover, they can be degraded by soil microorganisms and sorbed by SOM. All of them can be derived from both petrogenic and pyrogenic sources and therefore may be representative compounds for the PAH group.

The analytical procedure for TE-PAHs was fully described by Maliszewska-Kordybach et al. [1,2] and Ukalska-Jaruga et al. [8,24]. The analytical steps included extraction of ground soil samples (grain size ≤ 0.10 mm) with dichloromethane in an ASE200 Accelerated Solvent Extractor (Dionex Co., Sunnyvale, CA, USA). Before extraction, the samples were mixed with 2 g of diatomaceous earth dried at 550 °C and spiked with 10 μL of a recovery standard containing five deuterated PAHs (PAH-31, Dr. Ehrenstorfer GmbH, Augsburg, Germany). Clean-up of the concentrated extract was performed using hexane followed by hexane/dichloromethane (2:3 *v*/*v*) elution on activated silica gel (16 h at 135 °C). 

PB-PAHs were extracted using hydroxypropyl-β-cyclodextrin (HPCD) solution according to ISO/TS 16751:2018. HPCD was applied as a complexing agent encapsulating PAH free molecules desorbing from soil to the extracting solution. Briefly, a dry soil sample of 4 ± 1 g was placed in the 100 mL centrifuge vial, and then 40 mL of 0.1 mol dm^−3^ HPCD solution was added. Extraction was carried out using the horizontal shaker (SM-30, Edward Bühler GmbH, Bodelshausen, Germany) equipped with a thermostat (TM-30, Edward Bühler GmbH, Bodelshausen, Germany) and a minichiller (Huber) in the dark, at 20 °C for 20 h at an amplitude of 150–180 rpm. After extraction, the soil/HPCD mixtures were centrifuged, then a certain amount of water phase (>30 mL) was collected in the separatory funnel and mixed with petroleum ether for liquid/liquid extraction. The organic phase was collected, evaporated (Vario CVC 2000, Vacuumbrand), concentrated in n-hexane and subjected to GC-MS/MS analysis (Agilent 7890B GC system; Agilent Tech., Santa Clara, CA, USA equipped with an Agilent 7000C detector and Agilent 7693 Autosampler). Soil samples were extracted in duplicates. The blank sample procedure was included in all extraction series.

The concentrations of TE-PAHs and PB-PAHs were determined by gas chromatography triple mass spectrometry on an Agilent 7890B GC system (Agilent Tech., Santa Clara, CA, USA), equipped with an Agilent 7000C detector and an Agilent 7693 Autosampler. Sample analyses were performed in multiple reaction monitoring (MRM) mode with diagnostic ions as recommended in ISO 22892:2006. Quality control measures included analysis of a certified reference material (CRM 131, ANAB Accredited Tested Laboratory), duplicate matrix samples, and a solvent blank sample. The precision expressed as a relative standard deviation (RSD) was in the range from 5% to 12%, and the recovery for individual compounds from CRM 131 was within 62% and 84%. The limit of quantification (LoQ) for individual PAH compounds ranged from 0.02 to 2.10 µg kg^−1^, while the limit of detection (LoD) was within the 0.01 to 0.81 µg kg^−1^ range.

### 3.4. Determination of SOM and Its Fractions 

The SOM fractionation was based on the isolation of the HS individual fractions, comprising FA, HA, and HN. The procedure was carried out using an adapted ISO 12782-4 method (ISO 12782-4, 2012, [47]), approved by the International Humic Substances Society [27]. The soil samples were acidified by 0.1 M HCl at pH 1 (1:10 *w*/*v* soil to solution ratio), shaken for 1 h in a laboratory shaker, and then centrifuged. The supernatant containing the first extract of the low molecular fulvic fraction was removed from the residue by decantation and discarded. The remains of the soil sample were neutralized with 0.1 M NaOH and then extracted with 1 M NaOH (1:10 *w*/*v* at pH > 12) overnight by continuous shaking. The decanted supernatant containing the mixture of FA and HA was acidified with 6 M HCl to gravimetrically precipitate the HA. The solutions of FA and HA were purified on DAX-8 polymer resin and analyzed for organic carbon content by a C–N analyzer (Multi N/C 2100/2100S Jena Analytics). The HN content was determined in the remaining soil solid residue after FA and HA extraction. Concentrations of organic carbon in the HN fractions were determined by a C–N analyzer (Vario Macro Cube, Elementar Analysensysteme GmbH, Langenselbold, Germany).

### 3.5. Statistical Analysis

Statistica software (Dell Statistica, version 13.3, TIBCO Software Inc. Greenwood Village, CO, USA) was used for statistical analysis. Basic statistical parameters such as mean, median, extreme values (min, max), lower quartile (LQ), upper quartile (UQ), and coefficient of variation (CoV) were calculated. The chi-squared test was applied to check the normal distribution of the results, and the principal component analysis (PCA) was used to provide an overview of the groups of PAHs and soil organic matter fractions. The PCA used an orthogonal transformation to convert a set of observations of possibly correlated variables into a set of values of linearly uncorrelated variables called principal components. The number of significant PCA factors was determined based on the scree plot. All analyzed relationships between variables marked as statistically significant were measured as *p* < 0.05.

## 4. Conclusions

The assessment of the availability and persistence of PAHs in soils subjected to long-term anthropopressure from industrial and domestic sources indicated that the behavior of contaminants depends on the SOM content and its fractional composition. The results pointed out that availability of PAHs is promoted by labile organic matter fractions including FA and HA, while their persistence depends on the relatively stable carbon forms, such as HN. Therefore, the higher contribution of humic acids in total SOM content raises the potential mobility and bioavailability/bioaccessibility of hydrocarbons to living organisms. Therefore, the processes of SOM transformations (e.g., expressed by HA/FA proportions) significantly affect the sorption and form of PAHs in soils. This indicates that the transformation process of organic matter constitutes an important factor in the intra-soil processing responsible for the retention of PAHs in soils. The more advanced the humification processes, the greater persistence of PAHs and the lower the availability, causing lower bioaccumulation, biodegradation, and toxicity of PAHs.

## Figures and Tables

**Table 1:** Concentrations of total extractable PAHs (TE-PAHs), potentially available PAHs (PB-PAHs), and residual PAHs (RE-PAHs) in soil samples (*n* = 20). Data expressed in µg kg^−1^.

	Min	Max	Med	Aver	LQ	UQ	CoV (%)
TE-FLA	84.6	1220.1	445.1	486.7	365.7	565.5	55.8
TE-PYR	50.3	948.3	326.1	367.9	271.2	379.7	63.1
TE-BaA	15.4	725.8	115.7	152.3	98.2	152.9	99.3
TE-CHR	43.2	837.0	154.2	213.2	145.6	205.7	83.6
∑4 TE-PAHs	193.5	3169.5	1065.1	1220.2	887.6	1271.2	63.1
PB-FLA	2.0	59.8	9.0	14.2	6.9	13.6	104.5
PB-PYR	0.8	59.3	15.8	18.1	2.7	26.5	93.5
PB-BaA	0.0	52.0	0.0	7.8	0.0	2.5	212.7
PB-CHR	0.0	84.1	3.3	16.2	0.7	23.2	153.5
∑4 PB-PAHs	4.3	226.4	37.8	56.2	9.9	79.4	110.8
RE-FLA	77.3	1209.4	440.2	472.5	340.1	526.9	57.3
RE-PYR	32.4	945.4	321.1	349.8	223.2	376.0	67.7
RE-BaA	13.4	725.8	110.3	144.5	82.4	152.9	104.1
RE-CHR	23.2	837.0	151.7	197.0	119.1	186.2	91.1
∑4 RE-PAHs	148.6	3164.7	1042.5	1163.9	769.2	1178.8	66.3

PAHs—polycyclic aromatic hydrocarbons, TE—total extractable PAHs fraction, PB—potential available PAHs fraction, RE—nonavailable/residual PAHs fraction, FLA—fluoranthene, PYR—pyrene, BaA—benz(a)anthracene, CHR—chrysene; Min—the lowest content of the test compound, above the detection limit, Max—the highest content of the analyzed compounds, Med—median, Aver—average, Lower Q—lower quartile, Upper Q—upper quartile, CoV—coefficient of variation, *n*—number of samples.

**Table 2:** Soil organic matter fractional composition in soils (*n* = 20). Data expressed in g kg^−1^.

	Min	Max	Med	Aver	LQ	UQ	CoV (%)
FA	0.4	7.5	1.7	2.5	1.5	3.2	68.3
HA	0.6	13.0	6.9	7.7	5.2	11.5	43.2
HN	0.9	122.9	4.3	16.4	2.9	10.3	192.4
HA/FA	0.3	18.2	3.7	4.1	3.3	4.0	86.1
(FA + HA)/HN	0.1	8.1	1.9	2.6	1.1	3.3	89.5

FA—fulvic acids, HA—humic acids, HN—humins; Min—the lowest value of the parameter, Max—the highest value of the parameter, Med—median, Aver—average, LQ—lower quartile, UQ—upper quartile, CoV—coefficient of variation.

**Table 3:** Relationships between polycyclic aromatic hydrocarbon (PAH) operational fractions and soil organic matter (SOM) fractions in soils (*n* = 20).

	FA	HA	HN	HA/FA	(FA+HA)/HN	TOC
PB-FLA	0.50 *	0.56 *	−0.13	−0.23	−0.02	−0.05
PB-PYR	0.14	0.55 *	−0.14	−0.23	−0.30	−0.12
PB-BaA	0.41	0.55 *	0.19	−0.17	−0.21	0.25
PB-CHR	0.73 *	0.32	0.10	−0.28	−0.31	0.17
∑4 PB-PAHs	0.56 *	0.52 *	0.02	−0.28	−0.26	0.09
TE-FLA	0.57 *	0.59 *	0.41	0.06	0.00	0.47 *
TE-PYR	0.20	0.33	0.68 *	0.50 *	−0.11	0.70 *
TE-BaA	−0.11	0.10	0.81 *	0.82 *	−0.26	0.78 *
TE-CHR	0.04	0.21	0.83 *	0.74 *	−0.24	0.82 *
∑4 TE-PAHs	0.21	0.34	0.70 *	0.50 *	−0.14	0.72 *
RE-FLA	0.54 *	0.56 *	0.69 *	0.07	0.00	0.48 *
RE-PYR	0.18	0.32	0.68 *	0.51 *	−0.09	0.69 *
RE-BaA	−0.16	0.05	0.79 *	0.84 *	−0.23	0.76 *
RE-CHR	−0.06	0.17	0.81 *	0.77 *	−0.19	0.79 *
∑ RE-PAHs	0.17	0.31	0.75 *	0.52 *	−0.12	0.71 *

* Significant correlations; PAHs—polycyclic aromatic hydrocarbons, TE—total extractable PAHs fraction, PB—potential available PAHs fraction, RE—nonavailable/residual PAHs fraction, FLA—fluoranthene, PYR—pyrene, BaA—benz(a)anthracene, CHR—chrysene; FA—fulvic acids, HA—humic acids, HN—humins.

**Table 4:** Physicochemical properties of soils (*n* = 20).

	Min	Max	Med	Aver	LQ	UQ	CoV (%)
Clay (%)	0	6	1	1	0	2	125
Silt (%)	10	38	26	24	20	29	34
Sand (%)	57	90	73	75	69	80	12
pH in KCl	3.8	6.3	5.0	4.9	4.3	5.4	14.9
TOC (g kg^−1^)	8.0	130.0	13.8	27.3	11.5	22.4	120.6
TC (g kg^−1^)	10.0	128.2	15.7	29.9	13.1	24.4	112.9
TN (g kg^−1^)	0.8	6.8	1.2	1.9	1.1	1.9	80.8
TC:TN	11.4	35.9	12.7	14.1	12.0	13.6	37.5
CEC (cmol ( + )∙kg^−1^)	0.5	11.4	4.1	5.2	3.3	6.3	64.1

TOC—total organic carbon, TN—total nitrogen, TC—total carbon, CEC—cation exchange capacity; Min—the lowest value of the parameter, Max—the highest value of the parameter, Med—median, Aver—average, LQ—lower quartile, UQ—upper quartile, CoV—coefficient of variation.

**Table 5:** Chemical and physical properties of polycyclic aromatic hydrocarbons (PAHs) analyzed in the research.

Compound	Abbreviation	Molecular Weight (g mol^−1^)	Solubility in Water (mg L^−1^)	Log K_o/w_	Area (Å^2^)	Volume (Å^3^)
Fluoranthene	FLA	202.3	0.260	5.22	222.8	195.1
Pyrene	PYR	202.3	0.132	5.18	217.8	193.6
Benz(a)anthracene	BaA	228.3	0.011	5.91	250.3	222.1
Chrysene	CHR	228.3	0.002	5.91	246.9	221.5
